# Medical History from the Earliest Times

**Published:** 1892-10-01

**Authors:** E. T. Withington


### Medical History from the Earliest Times.

By E. T. Withington, M.A., M.B. Oxon.

*(Continued from page* 406, *Vol. XII.)*

### XXV.—NESTORIAN MEDICAL SCHOOLS.

The great importance of the Nestorians in the history of medicine must excuse a short incursion into the domain of theology. In the year 429 Nestorius, then recently appointed to the see of Constantinople, preached a series of sermons in which he declared that the title, "Mother of God," could not rightly be applied to the Blessed Virgin. We need not repeat his arguments; the whole tendency of the age was against him; he was accused, despite his protests, of separating the natures of Christ, and in 431 the Council of Ephesus pronounced the anathema of the Church against the Archbishop and all his followers. But the heresy still flourished in Syria and the East, and the Nestorians, debarred from attaining eminence in Church or State, devoted themselves, like the Jews at a somewhat later date, especially to medicine. A hospital, having, it is said, 300 beds, already existed at Edessa, and there they established a school of medicine and philosophy. Love of life conquered even the hatred of heresy, and Nestorian practitioners were to be found at the orthodox court of Byzantium, one of whom, Stephen of Edessa, appears to have become friend and physician to the lords both of Rome and Persia. But this could not long continue; the students of Edessa began to discuss theology, and in 489 the Emperor Zeno ordered the school to be closed, and the heretics to be driven from the Empire.

They were received in Persia by the liberal-minded Shah, Kobad, whose yet greater son, Chosroes Nushirvan, soon afterwards welcomed the last of the pagan philosophers, whom the edict of Justinian had expelled from the schools of Alexandria and Athens. The doctrine which the Nestorians had resisted was one singularly repugnant, as they understood it, both to Persians and Moslem: "I will cleave the skull of the blasphemer, who says the Eternal God has a mother!" is an exclamation attributed to a Mahometan conqueror; and this may have had something to do with the remarkable favour shown to those heretics, both by the Shahs and the Caliphs. Their skill in medicine, and their hostility to the orthodox Christians of the empire, would doubtless still further recommend them to the favour of their new rulers.

When the exiles arrived in Persia they found at Gondisapor a kind of school or university, but whether in the hands of Zoroastrians, or of descendants of the Persian Christians said to have been converted by St. Thomas the Apostle, we do not know. It may, indeed, have been of Greek origin, for, though the Arabic story that physicians sent by Aurelian to attend his sister, the wife of Sapor, settled there, is unfounded, the town was originally peopled by captives from the cities and armies of the Empire. In any case it rapidly became Nestorian, though the heretics, with remarkable toleration, allowed the pagan philosophers to take part in the teaching. As at Edessa, the medical faculty seems to have predominated, and there are even traces of clinical instruction, for we find the students of theology forbidden to "follow" the physicians, while those of medicine are urged not to omit the daily reading of religious books. Another chronicler is yet more explicit. All the Christian students, he says, began their day's work by reading the Psalms and Gospels, then the theologians betook themselves to the study of the commentators, while the medicals went to the hospital. The same applies to other Nestorian schools at Seleucia and elsewhere, in each of which medicine seems to have been studied, and a Catholic historian remarks with astonishment that the physicians and jurists had an equal vote with the bishops and presbyters in the election of the patriarch.

Meanwhile Nestorian Christianity spread rapidly in Persia, and its missionaries penetrated to the confines of China and the heart of India. It may have been information thus obtained which induced Nushirvan to send his own physician, Burzweih, to India, to examine into and report upon the state of medicine in that country. He brought back, not only medical works—probably those of Susruta and Charaka—but things yet more valuable, the game of chess, and the Hitopadesa, a collection of Hindu tales, said to lie at the base of the "Arabian Nights" and of half the romances of mediæval Europe. At a later period we even find Hindu physicians among the professors at Gondisapor.

Thus were gathered together in one spot the ancient knowledge of the East, the remains of Greek free-thought, and the most liberal-minded of the Christians, awaiting only the impulse of some fresher and more vigorous activity to take a new step forward in science. But the physicians and philosophers of Gondisapor would indeed have been astonished had they been told that this impulse was destined to come from the wandering hordes of the Arabian desert.

